# Supplementary material for: Discovery and application of insertion-deletion (INDEL) polymorphisms for QTL mapping of early life-history traits in Atlantic salmon
Source: BMC Genomics. 2010 Mar 8;11:156. doi: 10.1186/1471-2164-11-156 (PMC2838853; doi:10.1186/1471-2164-11-156)
Supplement: Additional file 2 — Information on developed 76 locus single-run INDEL panel in Atlantic salmon. Information on fluorescence labeling, primer concentrations, PCR pooling and links to alignments, INDEL motifs and GENESCAN (Burge and Karlin 1997) predictions of genes/exons are available in html format. [file 1471-2164-11-156-S2.ZIP › Additionalfile2/snpsummary905.html]

```
Cluster 108 Contig 5

prev  Summary    Contig List  next
```

Size of Consensus sequence = 1032

Number of sequences = 75

Minimum redundancy = 6

Key

A gi|117531413|gb|EG862858.1|EG862858 EST\_ssal\_eve\_55864 ssaleve thyroid Salmo salar cDNA Salmo salar cDNA clone ssal\_eve\_576\_054\_rev 5', mRNA sequence  
B gi|29319705|gb|CB507947.1|CB507947 ssalob508283 reproductive Salmo salar cDNA, mRNA sequence  
C gi|117832224|gb|EG904920.1|EG904920 EST\_ssal\_evf\_7585 ssalevf mixed\_tissue Salmo salar cDNA Salmo salar cDNA clone ssal\_evf\_508\_181\_rev 5', mRNA sequence  
D gi|117533556|gb|EG865001.1|EG865001 EST\_ssal\_eve\_57793 ssaleve thyroid Salmo salar cDNA Salmo salar cDNA clone ssal\_eve\_578\_300\_rev 5', mRNA sequence  
E gi|117546463|gb|EG877908.1|EG877908 EST\_ssal\_eve\_21478 ssaleve thyroid Salmo salar cDNA Salmo salar cDNA clone ssal\_eve\_529\_077\_rev 5', mRNA sequence  
F gi|117532496|gb|EG863941.1|EG863941 EST\_ssal\_eve\_56839 ssaleve thyroid Salmo salar cDNA Salmo salar cDNA clone ssal\_eve\_577\_177\_rev 5', mRNA sequence  
G gi|117832222|gb|EG904918.1|EG904918 EST\_ssal\_evf\_7584 ssalevf mixed\_tissue Salmo salar cDNA Salmo salar cDNA clone ssal\_evf\_508\_181\_fwd 3', mRNA sequence  
H gi|29312911|gb|CB501685.1|CB501685 ssalga508152 head Salmo salar cDNA, mRNA sequence  
I gi|117531659|gb|EG863104.1|EG863104 EST\_ssal\_eve\_56085 ssaleve thyroid Salmo salar cDNA Salmo salar cDNA clone ssal\_eve\_576\_172\_rev 5', mRNA sequence  
J gi|24337229|gb|CA037776.1|CA037776 ssallna015037 liver Salmo salar cDNA, mRNA sequence  
K gi|117430717|gb|EG762941.1|EG762941 EST\_ssal\_sjb\_968 ssalsjb mixed\_tissue Salmo salar cDNA Salmo salar cDNA clone ssal\_sjb\_006\_123\_rev 5', mRNA sequence  
L gi|117530104|gb|EG861745.1|EG861745 EST\_ssal\_eve\_54862 ssaleve thyroid Salmo salar cDNA Salmo salar cDNA clone ssal\_eve\_574\_300\_rev 5', mRNA sequence  
M gi|117435261|gb|EG767484.1|EG767484 EST\_ssal\_evd\_43519 ssalevd thymus Salmo salar cDNA Salmo salar cDNA clone ssal\_evd\_558\_153\_rev 5', mRNA sequence  
N gi|117855192|gb|EG927888.1|EG927888 EST\_ssal\_evf\_30256 ssalevf mixed\_tissue Salmo salar cDNA Salmo salar cDNA clone ssal\_evf\_540\_060\_rev 5', mRNA sequence  
O gi|117486328|gb|EG818545.1|EG818545 EST\_ssal\_evd\_20671 ssalevd thymus Salmo salar cDNA Salmo salar cDNA clone ssal\_evd\_526\_253\_fwd 3', mRNA sequence  
P gi|117489069|gb|EG821286.1|EG821286 EST\_ssal\_evd\_23138 ssalevd thymus Salmo salar cDNA Salmo salar cDNA clone ssal\_evd\_529\_381\_rev 5', mRNA sequence  
Q gi|117532027|gb|EG863472.1|EG863472 EST\_ssal\_eve\_56417 ssaleve thyroid Salmo salar cDNA Salmo salar cDNA clone ssal\_eve\_576\_341\_rev 5', mRNA sequence  
R gi|117455996|gb|EG788215.1|EG788215 EST\_ssal\_evd\_8712 ssalevd thymus Salmo salar cDNA Salmo salar cDNA clone ssal\_evd\_510\_148\_rev 5', mRNA sequence  
S gi|117468689|gb|EG800908.1|EG800908 EST\_ssal\_evd\_57600 ssalevd thymus Salmo salar cDNA Salmo salar cDNA clone ssal\_evd\_577\_263\_rev 5', mRNA sequence  
T gi|117484212|gb|EG816429.1|EG816429 EST\_ssal\_evd\_18765 ssalevd thymus Salmo salar cDNA Salmo salar cDNA clone ssal\_evd\_524\_029\_rev 5', mRNA sequence  
U gi|117530105|gb|EG861746.1|EG861746 EST\_ssal\_eve\_54863 ssaleve thyroid Salmo salar cDNA Salmo salar cDNA clone ssal\_eve\_574\_300\_fwd 3', mRNA sequence  
V gi|117532498|gb|EG863943.1|EG863943 EST\_ssal\_eve\_56840 ssaleve thyroid Salmo salar cDNA Salmo salar cDNA clone ssal\_eve\_577\_177\_fwd 3', mRNA sequence  
W gi|117453442|gb|EG785661.1|EG785661 EST\_ssal\_evd\_41879 ssalevd thymus Salmo salar cDNA Salmo salar cDNA clone ssal\_evd\_556\_042\_rev 5', mRNA sequence  
X gi|117497249|gb|EG829466.1|EG829466 EST\_ssal\_eve\_41812 ssaleve thyroid Salmo salar cDNA Salmo salar cDNA clone ssal\_eve\_556\_335\_rev 5', mRNA sequence  
Y gi|117504261|gb|EG836020.1|EG836020 EST\_ssal\_eve\_47711 ssaleve thyroid Salmo salar cDNA Salmo salar cDNA clone ssal\_eve\_564\_335\_rev 5', mRNA sequence  
Z gi|117859420|gb|EG932116.1|EG932116 EST\_ssal\_evf\_34062 ssalevf mixed\_tissue Salmo salar cDNA Salmo salar cDNA clone ssal\_evf\_545\_117\_rev 5', mRNA sequence  
a gi|24342426|gb|CA041506.1|CA041506 ssalplnb507219 gut Salmo salar cDNA, mRNA sequence  
b gi|117525056|gb|EG856783.1|EG856783 EST\_ssal\_eve\_50395 ssaleve thyroid Salmo salar cDNA Salmo salar cDNA clone ssal\_eve\_568\_237\_rev 5', mRNA sequence  
c gi|117491860|gb|EG824077.1|EG824077 EST\_ssal\_evd\_25650 ssalevd thymus Salmo salar cDNA Salmo salar cDNA clone ssal\_evd\_533\_147\_rev 5', mRNA sequence  
d gi|117533557|gb|EG865002.1|EG865002 EST\_ssal\_eve\_57794 ssaleve thyroid Salmo salar cDNA Salmo salar cDNA clone ssal\_eve\_578\_300\_fwd 3', mRNA sequence  
e gi|117862995|gb|EG935691.1|EG935691 EST\_ssal\_evf\_19690 ssalevf mixed\_tissue Salmo salar cDNA Salmo salar cDNA clone ssal\_evf\_524\_332\_rev 5', mRNA sequence  
f gi|117522256|gb|EG853983.1|EG853983 EST\_ssal\_eve\_14778 ssaleve thyroid Salmo salar cDNA Salmo salar cDNA clone ssal\_eve\_520\_025\_rev 5', mRNA sequence  
g gi|117859419|gb|EG932115.1|EG932115 EST\_ssal\_evf\_34061 ssalevf mixed\_tissue Salmo salar cDNA Salmo salar cDNA clone ssal\_evf\_545\_117\_fwd 3', mRNA sequence  
h gi|117507362|gb|EG839121.1|EG839121 EST\_ssal\_eve\_2101 ssaleve thyroid Salmo salar cDNA Salmo salar cDNA clone ssal\_eve\_501\_350\_rev 5', mRNA sequence  
i gi|117459738|gb|EG791957.1|EG791957 EST\_ssal\_evd\_12081 ssalevd thymus Salmo salar cDNA Salmo salar cDNA clone ssal\_evd\_515\_006\_rev 5', mRNA sequence  
j gi|117475176|gb|EG807395.1|EG807395 EST\_ssal\_evd\_27975 ssalevd thymus Salmo salar cDNA Salmo salar cDNA clone ssal\_evd\_536\_224\_rev 5', mRNA sequence  
k gi|117508621|gb|EG840380.1|EG840380 EST\_ssal\_eve\_3235 ssaleve thyroid Salmo salar cDNA Salmo salar cDNA clone ssal\_eve\_503\_161\_rev 5', mRNA sequence  
l gi|117517807|gb|EG849566.1|EG849566 EST\_ssal\_eve\_7002 ssaleve thyroid Salmo salar cDNA Salmo salar cDNA clone ssal\_eve\_508\_194\_rev 5', mRNA sequence  
m gi|117459679|gb|EG791898.1|EG791898 EST\_ssal\_evd\_12028 ssalevd thymus Salmo salar cDNA Salmo salar cDNA clone ssal\_evd\_514\_364\_rev 5', mRNA sequence  
n gi|85161774|gb|DW591378.1|DW591378 smus1-009AG02.y1\_#0537.2 DIAS\_SMUS Salmo salar cDNA 5', mRNA sequence  
o gi|59834446|gb|DN140129.1|DN140129 SGP266151 Atlantic salmon Head kidney cDNA library Salmo salar cDNA clone FN4-3469 5', mRNA sequence  
p gi|84974436|gb|DW472837.1|DW472837 SGP313702 Atlantic salmon Spleen cDNA library Salmo salar cDNA clone MI5-2904 5', mRNA sequence  
q gi|117504262|gb|EG836021.1|EG836021 EST\_ssal\_eve\_47712 ssaleve thyroid Salmo salar cDNA Salmo salar cDNA clone ssal\_eve\_564\_335\_fwd 3', mRNA sequence  
r gi|117427843|gb|EG760067.1|EG760067 EST\_ssal\_sjb\_4264 ssalsjb mixed\_tissue Salmo salar cDNA Salmo salar cDNA clone ssal\_sjb\_011\_089\_fwd 3', mRNA sequence  
s gi|117517808|gb|EG849567.1|EG849567 EST\_ssal\_eve\_7003 ssaleve thyroid Salmo salar cDNA Salmo salar cDNA clone ssal\_eve\_508\_194\_fwd 3', mRNA sequence  
t gi|117435260|gb|EG767483.1|EG767483 EST\_ssal\_evd\_43518 ssalevd thymus Salmo salar cDNA Salmo salar cDNA clone ssal\_evd\_558\_153\_fwd 3', mRNA sequence  
u gi|24339432|gb|CA039086.1|CA039086 ssalnwh007062 whole Salmo salar cDNA, mRNA sequence  
v gi|117855193|gb|EG927889.1|EG927889 EST\_ssal\_evf\_30257 ssalevf mixed\_tissue Salmo salar cDNA Salmo salar cDNA clone ssal\_evf\_540\_060\_fwd 3', mRNA sequence  
w gi|117459737|gb|EG791956.1|EG791956 EST\_ssal\_evd\_12080 ssalevd thymus Salmo salar cDNA Salmo salar cDNA clone ssal\_evd\_515\_006\_fwd 3', mRNA sequence  
x gi|117453443|gb|EG785662.1|EG785662 EST\_ssal\_evd\_41880 ssalevd thymus Salmo salar cDNA Salmo salar cDNA clone ssal\_evd\_556\_042\_fwd 3', mRNA sequence  
y gi|117488558|gb|EG820775.1|EG820775 EST\_ssal\_evd\_22679 ssalevd thymus Salmo salar cDNA Salmo salar cDNA clone ssal\_evd\_529\_139\_fwd 3', mRNA sequence  
z gi|117531660|gb|EG863105.1|EG863105 EST\_ssal\_eve\_56086 ssaleve thyroid Salmo salar cDNA Salmo salar cDNA clone ssal\_eve\_576\_172\_fwd 3', mRNA sequence  
A gi|117532026|gb|EG863471.1|EG863471 EST\_ssal\_eve\_56416 ssaleve thyroid Salmo salar cDNA Salmo salar cDNA clone ssal\_eve\_576\_341\_fwd 3', mRNA sequence  
B gi|117526344|gb|EG858071.1|EG858071 EST\_ssal\_eve\_51555 ssaleve thyroid Salmo salar cDNA Salmo salar cDNA clone ssal\_eve\_570\_083\_fwd 3', mRNA sequence  
C gi|117508620|gb|EG840379.1|EG840379 EST\_ssal\_eve\_3234 ssaleve thyroid Salmo salar cDNA Salmo salar cDNA clone ssal\_eve\_503\_161\_fwd 3', mRNA sequence  
D gi|117430718|gb|EG762942.1|EG762942 EST\_ssal\_sjb\_969 ssalsjb mixed\_tissue Salmo salar cDNA Salmo salar cDNA clone ssal\_sjb\_006\_123\_fwd 3', mRNA sequence  
E gi|117468688|gb|EG800907.1|EG800907 EST\_ssal\_evd\_57599 ssalevd thymus Salmo salar cDNA Salmo salar cDNA clone ssal\_evd\_577\_263\_fwd 3', mRNA sequence  
F gi|117455995|gb|EG788214.1|EG788214 EST\_ssal\_evd\_8711 ssalevd thymus Salmo salar cDNA Salmo salar cDNA clone ssal\_evd\_510\_148\_fwd 3', mRNA sequence  
G gi|117455651|gb|EG787870.1|EG787870 EST\_ssal\_evd\_8403 ssalevd thymus Salmo salar cDNA Salmo salar cDNA clone ssal\_evd\_509\_371\_fwd 3', mRNA sequence  
H gi|117507363|gb|EG839122.1|EG839122 EST\_ssal\_eve\_2102 ssaleve thyroid Salmo salar cDNA Salmo salar cDNA clone ssal\_eve\_501\_350\_fwd 3', mRNA sequence  
I gi|117489070|gb|EG821287.1|EG821287 EST\_ssal\_evd\_23139 ssalevd thymus Salmo salar cDNA Salmo salar cDNA clone ssal\_evd\_529\_381\_fwd 3', mRNA sequence  
J gi|117497248|gb|EG829465.1|EG829465 EST\_ssal\_eve\_41811 ssaleve thyroid Salmo salar cDNA Salmo salar cDNA clone ssal\_eve\_556\_335\_fwd 3', mRNA sequence  
K gi|117522255|gb|EG853982.1|EG853982 EST\_ssal\_eve\_14777 ssaleve thyroid Salmo salar cDNA Salmo salar cDNA clone ssal\_eve\_520\_025\_fwd 3', mRNA sequence  
L gi|117475177|gb|EG807396.1|EG807396 EST\_ssal\_evd\_27976 ssalevd thymus Salmo salar cDNA Salmo salar cDNA clone ssal\_evd\_536\_224\_fwd 3', mRNA sequence  
M gi|117491861|gb|EG824078.1|EG824078 EST\_ssal\_evd\_25651 ssalevd thymus Salmo salar cDNA Salmo salar cDNA clone ssal\_evd\_533\_147\_fwd 3', mRNA sequence  
N gi|117525057|gb|EG856784.1|EG856784 EST\_ssal\_eve\_50396 ssaleve thyroid Salmo salar cDNA Salmo salar cDNA clone ssal\_eve\_568\_237\_fwd 3', mRNA sequence  
O gi|117489328|gb|EG821545.1|EG821545 EST\_ssal\_evd\_23371 ssalevd thymus Salmo salar cDNA Salmo salar cDNA clone ssal\_evd\_530\_119\_fwd 3', mRNA sequence  
P gi|117484211|gb|EG816428.1|EG816428 EST\_ssal\_evd\_18764 ssalevd thymus Salmo salar cDNA Salmo salar cDNA clone ssal\_evd\_524\_029\_fwd 3', mRNA sequence  
Q gi|117531414|gb|EG862859.1|EG862859 EST\_ssal\_eve\_55865 ssaleve thyroid Salmo salar cDNA Salmo salar cDNA clone ssal\_eve\_576\_054\_fwd 3', mRNA sequence  
R gi|50134373|gb|CO469589.1|CO469589 SGP262114 Atlantic salmon Eye cDNA library Salmo salar cDNA clone OY4-0911 5', mRNA sequence  
S gi|117463384|gb|EG795603.1|EG795603 EST\_ssal\_evd\_15363 ssalevd thymus Salmo salar cDNA Salmo salar cDNA clone ssal\_evd\_519\_190\_fwd 3', mRNA sequence  
T gi|117862996|gb|EG935692.1|EG935692 EST\_ssal\_evf\_19691 ssalevf mixed\_tissue Salmo salar cDNA Salmo salar cDNA clone ssal\_evf\_524\_332\_fwd 3', mRNA sequence  
U gi|117546464|gb|EG877909.1|EG877909 EST\_ssal\_eve\_21479 ssaleve thyroid Salmo salar cDNA Salmo salar cDNA clone ssal\_eve\_529\_077\_fwd 3', mRNA sequence  
V gi|117505928|gb|EG837687.1|EG837687 EST\_ssal\_eve\_9340 ssaleve thyroid Salmo salar cDNA Salmo salar cDNA clone ssal\_eve\_512\_243\_fwd 3', mRNA sequence  
W gi|117526601|gb|EG858328.1|EG858328 EST\_ssal\_eve\_51787 ssaleve thyroid Salmo salar cDNA Salmo salar cDNA clone ssal\_eve\_570\_205\_fwd 3', mRNA sequence

8 SNPs detected

A B C D E F G H I J K L M N O P Q R S T U V W X Y Z a b c d e f g h i j k l m n o p q r s t u v w x y z A B C D E F G H I J K L M N O P Q R S T U V W  cosegregation weighted

285 G G G G G G G G G G A G G G G G G A A G G G G G G G A G G G G G G G G G G G G . . . G A G G . G G G G . G G G A A A G G G G . G G G G G . . G G . G .   5/8 54.17
298 A A A A A A A A A A A A - - A A A A A A A A A A A A A A A A - - A A A A A - - . . . A - - - . - A A A A A A A - - - A A A A . A A A A A . . A - . A A   1/8 11.17
443 T T T T T T T T T T G T T T T T T G G T T T T T T T G T T T T T T T T T T T T T . T T G T T T T T T T T T T T G G G T T T T T T T T T T T . T T T T T   5/8 60.83
466 . - - - - - - - - - T - - - - - - T T - - - - - - - T - - - - - - - - - - - - - . - - T - - - - - - - - - - - T T T - - - - - - - - - - - . - - - - -   5/8 60.00
467 . - - - - - - - - - T - - - - - - T T - - - - - - - T - - - - - - - - - - - - - . - - T - - - - - - - - - - - T T T - - - - - - - - - - - . - - - - -   5/8 60.00
468 . - - - - - - - - - A - - - - - - A A - - - - - - - A - - - - - - - - - - - - - . - - A - - - - - - - - - - - A A A - - - - - - - - - - - . - - - - -   5/8 60.00
997 . . . . . . . . . . . . . . . . . . . . . . . . . . . . . . . . . . . . . . . . . . C C C . . C C . C C C C G G C C C G G G G G G G . G G G G G G G G   1/8 4.83
999 . . . . . . . . . . . . . . . . . . . . . . . . . . . . . . . . . . . . . . . . . . . . . . . . . . . . . . C C . . . C C A A C C C . C A A A A A A A   1/8 2.83
